# Immunological response in mice bearing LM3 breast tumor undergoing Pulchellin treatment

**DOI:** 10.1186/1472-6882-12-107

**Published:** 2012-07-24

**Authors:** Djamile Cordeiro de Matos, Lívia Carolina Abreu de Ribeiro, Aline Tansini, Lucas Souza Ferreira, Marisa Campos Polesi Placeres, Lucas Luis Colombo, Iracilda Zeppone Carlos

**Affiliations:** 1 Laboratory of Clinical Immunology, School of Pharmaceutical Sciences, São Paulo State University, São Paulo, Brazil; 2 Institute of Oncology Angel H. Roffo, University of Buenos Aires, Buenos Aires, Argentina; 3 Faculdade de Ciências Farmacêuticas, Universidade Estadual Paulista, Rua Expedicionários do Brasil 1601, 14801-902, Araraquara, SP, Brazil

**Keywords:** Pulchellin, Breast cancer, Cytokines, Immune system

## Abstract

**Background:**

Ribosome-inactivating proteins (RIP) have been studied in the search for toxins that could be used as immunotoxins for cancer treatment. Pulchellin, a type 2 RIP, is suggested to induce immune responses that have a role in controlling cancer.

**Methods:**

The percentage of dendritic cells and CD4^+^ and CD8^+^ T cells in the spleen (flow cytometry), cytokines’ release by PECs and splenocytes (ELISA) and nitric oxide production by PECs (Griess assay) were determined from tumor-bearing mice injected intratumorally with 0.1 ml of pulchellin at 0.75 μg/kg of body weight. Statistical analysis was performed by one-way ANOVA with Tukey’s post hoc test.

**Results:**

Pulchellin-treated mice showed significant immune system activation, characterized by increased release of IFN-γ and Th2 cytokines (IL-4 and IL-10), while IL-6 and TGF-β levels were decreased. There was also an increase in macrophage’s activation, as denoted by the higher percentage of macrophages expressing adhesion and costimulatory molecules (CD54 and CD80, respectively).

**Conclusions:**

Our results suggest that pulchellin is promising as an adjuvant in breast cancer treatment.

## Background

Tumor cells are confronted with immune cells at each stage of malignancy in the host, within the tumor, in the blood and lymph circulation and in metastatic lesions [1]. Cancer immunotherapy aims to stimulate the immune system to destroy tumors, i.e. by enhancing the production of cytokines and other immune mediators that might act to eliminate the tumor cells.

Pulchellin is a type 2 ribosome-inactivating protein (RIP) isolated from the seeds of *Abrus pulchellus tenuiflorus*. Type 2 RIPs, like ricin and abrin, are heterodimeric proteins composed of an enzymatic A-chain covalently linked by a single disulfide bond to a B-chain. The A-chain has the toxic rRNA-specific N-glycosidase activity, and the B-chain has lectin activity towards specific carbohydrate moieties present on the surfaces of mammalian cells. Pulchellin has an affinity for β-D-galactose [2], a carbohydrate that is present on the surface of many eukaryotic cells. This feature enables this RIP to bind to glycoproteins and glycolipids on the cell surface, after which it is internalized by different mechanisms [3]. Several studies have reported the mechanisms of pulchellin interaction with membrane model systems [4] and the endocytosis machinery in K-562 cells [2], but there have been no reports of the immunological activities of this protein.

In the present study, we investigated the immune response in tumor-bearing mice treated with pulchellin. A high production of IFN-γ was observed, possibly responsible for the observed macrophage activation, including nitric oxide and TNF-α production and an enhanced expression of adhesion and costimulatory molecules; the increased production of the cytokines IL-10 and IL-4, but decreased IL-6 and TGF-β production, was also observed.

## Methods

### Materials

Native pulchellin was isolated [5] and kindly provided by Dr. Ana Paula Ulian de Araújo from the Physics Institute of São Carlos – USP, Brazil.

### Cell culture

The tumor cell line LM3, derived from a murine mammary adenocarcinoma that spontaneously arose in BALB/c mice, was kindly provided by Dr. Lucas L. Colombo from Angel H. Roffo Institute, Buenos Aires, Argentina. Cells were maintained in MEM medium (Gibco, BRL) supplemented with 3 mM L-glutamine, 80 μg/ml gentamycin and 5% heat-inactivated fetal calf serum (FCS) at 37°C in a humidified 5% CO_2_ air atmosphere. Cells were detached by the trypsinization (0.25% trypsin and 0.02% EDTA, in Ca^2+^- and Mg^2+^-free PBS) of confluent monolayers. The medium was replaced three times per week. Cell viability was assayed using a Trypan blue exclusion test, and the absence of mycoplasma was confirmed by the Hoechst method.

### Animals

Female BALB/c mice (2-3-month-old) weighing around 20 g were purchased from the animal facility of UNICAMP, São Paulo, Brazil. Sterilized water and food were provided *ad libitum*. All animal procedures were performed in accordance with the regulations of the Research Ethics Committee (# 28/2008), Faculty of Pharmaceutical Sciences, UNESP, São Paulo, Brazil.

### Experimental groups

LM3 cells (1 × 10^6^ cells) were injected subcutaneously in the flanks of 2-3-month-old female BALB/c mice. After 20 days, LM3 tumor-bearing mice were injected intratumorally with 0.1 ml of pulchellin at 0.75 μg/kg of body weight (10 times the *in vitro* IC_50_ concentration) (group P) or phosphate buffered saline (group T). The mice in group N did not receive treatment (healthy mice). The mice were sacrificed after 7 days of treatment.

### Peritoneal macrophages

Thioglycollate-elicited peritoneal exudate cells (PEC) were harvested from group P, group T and group N mice using 5.0 ml of sterile PBS, pH 7.4. The cells were washed twice by centrifugation at 200 ***g*** for 5 min at 4°C and resuspended in the appropriate medium for each test.

### Isolation of splenic lymphocytes

Spleens were resected from group P, group T and group N mice under sterile conditions and macerated to produce single cell suspensions. After red blood cell lysis, the cells were washed twice by centrifugation at 200 ***g*** for 5 min at 4°C and resuspended in the appropriate medium for each test.

### Flow cytometry analysis

The cells were adjusted to a concentration of 1x10^6^ cells/ml in PBS containing 1% BSA. Peritoneal exudate cells were stained with the following fluorescent dye-conjugated monoclonal antibodies (Mab) from BD Biosciences: CD11b fluorescein isothiocyanate (FITC), CD54 phycoerythrin (PE) and CD80 allophycocyanin (APC). Spleen cells were stained with the following fluorescent dye-conjugated monoclonal antibodies (Mab), also from BD Biosciences: CD3 FITC, CD4 PE, CD8a peridinin chlorophyll cyanine 5.5 (PerCP-Cy5.5), CD11c FITC, CD25 APC, CD86 APC and I-Ad/I-Ed (MHC II) PE. Corresponding IgG isotypes were used as controls to account for non-specific binding (BD Biosciences). Prior to cell staining, non-specific binding sites were blocked with mouse BD Fc Block (BD Biosciences). Cell surface markers were stained for 30 min at 4°C, washed with staining buffer (PBS containing 1% BSA) and then fixed in 1% paraformaldehyde at 4°C until analysis. Flow cytometry data were acquired using a FACSCanto instrument (BD Immunocytometry System, USA) and analyzed using FACSDiva Software (BD Biosciences).

### Measurement of nitric oxide (NO) production

NO production was determined by assaying the nitrite levels of the culture supernatants using the Griess reagent. PEC (adherent cells) at 5x10^6^ cells/ml in RPMI-1640 medium supplemented with 2x10^-5^ M 2-mercaptoethanol, 100 U/mL penicillin, 100 U/ml streptomycin, 2 mM L-glutamine and 5% FCS (complete RPMI) were incubated for 24 h at 37°C in a 7.5% CO_2_ atmosphere. Cell-free supernatant (50 μl) was mixed with 50 μl Griess reagent (0.1% sulfanilamide, 3% phosphoric acid and 0.1% naphthyl ethylenediamine) and incubated at room temperature for 10 min in the dark. After incubation, absorbance was read at 540 nm on a microplate reader (Multiskan, Labsystems, Finland). The nitrite concentration was calculated based on a standard curve. The results were expressed in μmols/ml [6].

### Measurement of cytokines production

The levels of IL-6 and TNF-α in the PEC culture supernatants and IL-4, IL-10, IFN-γ and TGF-β in the splenocytes culture supernatants were determined by a sandwich immunoassay kit (BD Biosciences Pharmingen, USA) in 96-well microplates performed according to the manufacturer’s instructions. Absorbance was read at 450 nm on a microplate reader (Multiskan Ascent, Labsystems, Finland) within 30 min of stopping the reaction, and cytokine concentrations were calculated from a curve of known concentrations of each cytokine standard. The results were expressed in pg/ml.

### Data analysis

The results are expressed as the means ± S.D. Each experiment was performed at least five times. One-way ANOVA with Tukey’s post hoc test was performed using GraphPad InStat version 3.0 for Windows 95, Graph-Pad Software, San Diego, California, U.S.A. Values of p < 0.05 were considered statistically significant.

## Results

### Flow cytometry analysis

Macrophage cell surface markers were used to evaluate peritoneal cells, lymphocytes and dendritic spleen cells. We observed that untreated tumor-bearing mice (group T) presented an increase in the numbers of mature dendritic cells (CD11c^+^CD86^+^ and CD11c^+^MHCII^+^, p < 0.05 and p < 0.01, respectively) and CD4^+^CD25^+^ cells (p < 0.01), accompanied by a reduction of total CD4^+^ and CD8^+^ cells (p < 0.001) when compared to control healthy mice (group N). Pulchellin treatment (group P) promoted an enhancement of adhesion (CD54) and costimulatory (CD80) molecules expression in peritoneal macrophages (p < 0.01), a significant reduction in the number of CD4^+^CD25^+^ Treg cells (p < 0.01) and non-significant reduction in the number of CD8 cells and dendritic cells (p > 0.05) relative to the tumor control group (T group) (Table 1).

**Table 1:** Percentage of macrophages (peritoneum), dendritic cells and lymphocytes (both from spleen) from mice of the different treatment groups

	**Pulchellin (P group)**	**Tumor control (T group)**	**Healthy mice (N group)**
% CD11b^+^CD54^+^	35.8 ± 2.6^##^***	24.7 ± 2.2	23.1 ± 1.4
% CD11b^+^CD80^+^	5.3 ± 1.3^##^**	1.1 ± 0.4	1.5 ± 0.2
% CD11c^+^MHCII^+^	1.5 ± 0.1^##^	2.8 ± 0,5**	1.2 ± 0.1
% CD11c^+^CD86^+^	0.2 ± 0.1^#^	0.4 ± 0.1*	0.2 ± 0.1
%CD3^+^CD4^+^	9.0 ± 0.3***	8.5 ± 0.2***	17.6 ± 1.0
% CD4^+^CD25^+^	0.1 ± 0.1^##^	1.0 ± 0.3**	0.1 ± 0.1
% CD3^+^CD8^+^	2.6 ± 0.4***	2.5 ± 0.4***	6.7 ± 0.4

Data are represented as the mean ± SEM. Statistical significance was evaluated by Tukey post hoc test. * p < 0.05, ** p < 0.01 and *** p < 0.001 vs. healthy group; # p < 0.05 and ## p < 0.01 vs. tumor control group.

### Measurement of nitric oxide and cytokine production

**Figure 1:**
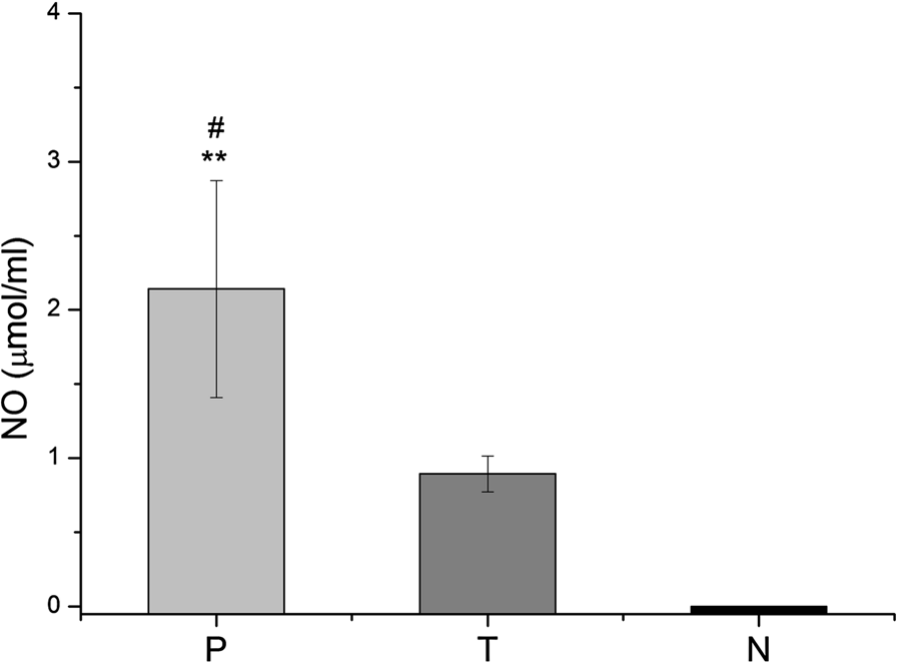
**NO Synthesis by Peritoneal Macrophages. Adherent cells (5x10**^**6 **^**cells ml**^**-1**^**) were incubated for 24 h with RPMI-1640 culture medium.** Cell-free supernatant was mixed with the Griess reagent and the resulting reaction was read in a spectrophotometer. P (tumor-bearing mice treated with pulchellin at 0.75 μg/kg of body weight), T (tumor-bearing mice treated with saline solution) and N (untreated healthy mice). One-way ANOVA with Tukey’s post test was performed. ** *p* < 0.01 *vs*. N group; ^#^ p < 0.05 vs. T group.

**Figure 2:**
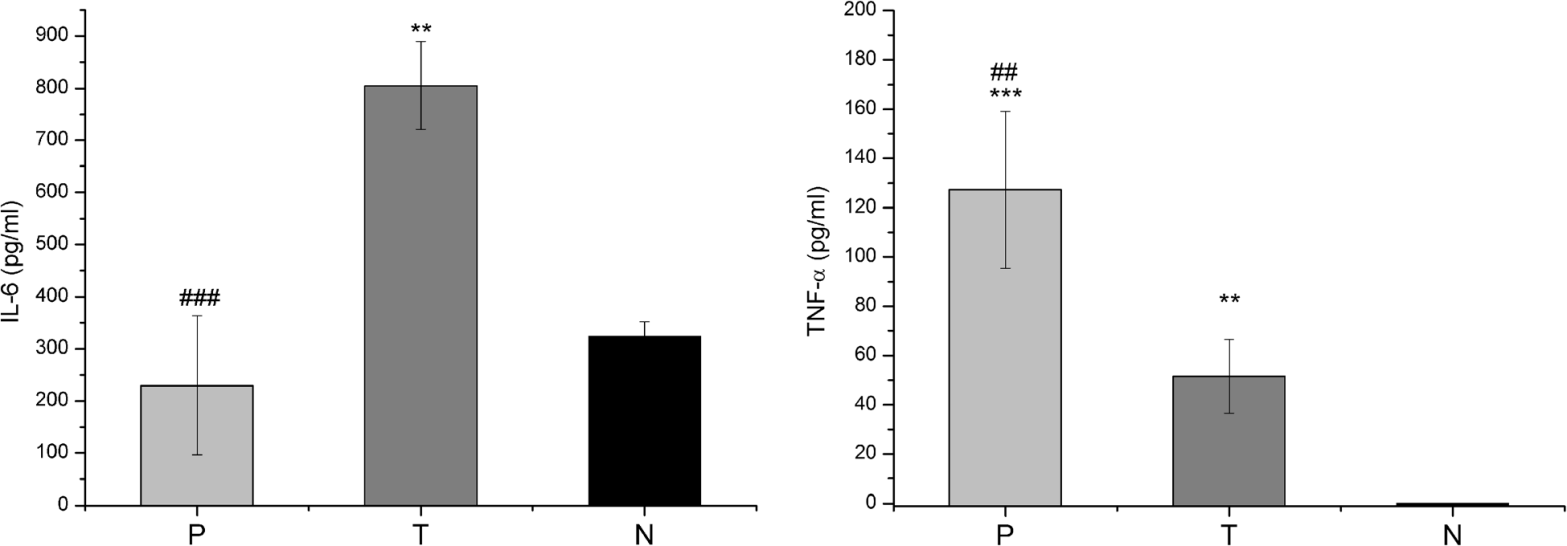
**IL-6 and TNF-α synthesis by PEC cells. For the cytokine immunoassays, adherent cells (5x10**^**6 **^**cells ml**^**-1**^**) were incubated for 24 h with RPMI-1640 culture medium.** Cell-free supernatant was assayed for IL-6 and TNF-α by ELISA according to the manufacturer’ instructions. P (tumor-bearing mice treated with pulchellin at 0.75 μg/kg of body weight), T (tumor-bearing mice treated with saline solution) and N (untreated healthy mice). One-way ANOVA with Tukey’s post test was performed. ** *p* < 0.01, *** p < 0.001 *vs*. N group; ^##^ p < 0.01 vs. T group.

**Figure 3:**
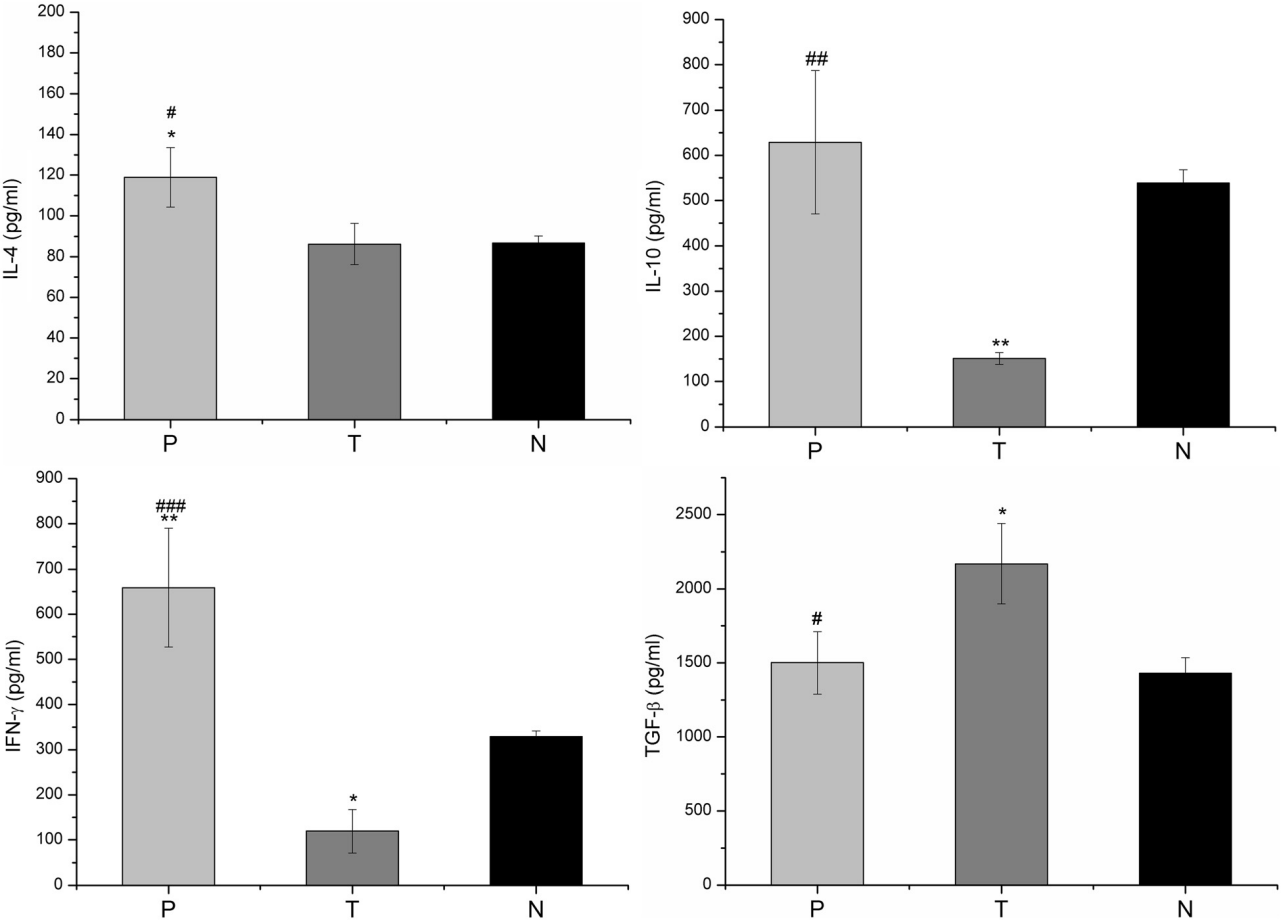
**IL-4, IL-10, IFN-γ and TGF-β synthesis by spleen cells. For the cytokine immunoassay, spleen cells (5x10**^**6 **^**cells ml**^**-1**^**) were incubated for 24 h with RPMI-1640 culture medium.** Cell-free supernatant was assayed for IL-4, IL-10, IFN-γ and TGF-β by ELISA according to the manufacturer’ instructions. P (tumor-bearing mice treated with pulchellin at 0.75 μg/kg of body weight), T (tumor-bearing mice treated with saline solution) and N (untreated healthy mice). One-way ANOVA with Tukey’s post test was performed. * *p* < 0.05, ** p < 0.01 *vs*. N group; ^#^ p < 0.05, ^##^ p < 0.01 and ^###^ p < 0.001 vs. T group.

In order to evaluate the immune response in mice that received an intratumoral administration of pulchellin, the production of nitric oxide and cytokines in the cell culture supernatants of PEC and spleen cells was determined. Analysis of the PEC cells from pulchellin-treated tumor-bearing mice revealed a higher production of NO than that of the untreated tumor-bearing or healthy mice (p < 0.05 and p < 0.01, respectively) after 24 h of culture (Figure 1). The immune cells from pulchellin-treated mice also produced statistically higher concentrations of TNF-α by PEC cells, and IL-4, IFN-γ and IL-10 by splenocytes, and lower concentrations of IL-6 (PEC cells) and TGF-β (splenocytes), than the cells from tumor control mice (Figures 2 and 3).

## Discussion

In the last several years, researchers have demonstrated the important role of the immune system in the control or promotion of tumor development [7]. Pulchellin, a type 2 RIP isolated from *Abrus pulchellus tenuiflorus’* seeds, is a powerful toxin, structurally similar to abrin and ricin. Although the anti-tumor activities and immunomodulatory effects of abrin and ricin have been studied [8-10], there is no such published data for pulchellin.

In this study, we evaluated the production of some immune mediators and the expression of surface markers in different immune cells obtained from tumor-bearing mice treated or not with pulchellin. We observed greater production of IFN-γ by spleen cells from group P than in those from group T. Since we couldn't detect IL-12 production by PECs from group P (data not shown), it can be suggested that the increased IFN-γ production found in this group is most likely the cause of the enhanced expression of adhesion (CD54) and costimulatory (CD80) molecules and the production of TNF-α and NO by macrophages [11]. In some cases, production of IFN-γ can be triggered in the absence of IL-12 stimulation, when there is enough antigen levels to keep a sustained TCR activation with subsequent fosforilation of the mitogen-activated Erk kinase protein, resulting in IFN-γ-producing Th1 cells [12].

We also observed less activation of dendritic cells from the spleens of group P mice than in mice from group T. A group of researchers have already reported a similar phenomenon when they treated immature human monocyte-derived dendritic cells with ricin (type II RIP), resulting in decreased expression of MHC II and CD86 in these cells. This may explain why pulchellin treatment does not significantly change the percentage of CD4^+^ and CD8^+^ T cells in comparison to group T [13].

A reduction of CD4^+^CD25^+^ cells, as well as of TGF-β and IL-6 production, was observed in group P compared to group T. IL-6 is known to promote tumor growth by upregulating anti-apoptotic and pro-angiogenic proteins in tumor cells [14], and TGF-β is known to support tumor progression by promoting cell invasion and dissemination to distant sites, enhancing angiogenesis and mediating the immune evasion of tumor cells [15]. Besides that, IL-6 is known to promote, together with TGF- β, naive T cells differentiation into Th17 cells [16], which are found in greater quantities in mice as breast cancer progresses, reaching their highest levels at the later stages of the disease [17]. CD4^+^CD25^+^ cells are naturally occurrbca03ing Tregs and are recognized as a major subset among the immune cells that are responsible for peripheral immune self-tolerance. The accumulation of CD4^+^CD25^+^ Tregs can result in tumor growth through the suppression of anticancer immunity [18]. Consequently, the downregulation of these Tregs and cytokines by pulchellin treatment might improve antitumor immunity.

The increase in IL-10 and IL-4 release on group P animals may enhance the host immune response against the tumor and aid in the control of tumor growth. IL-4 also plays a role in the immune response against various tumors, including breast cancer, by inhibiting tumor angiogenesis [19]. Meanwhile, IL-10 directly activates and expands the antigen-specific IFN-γ-producing effector CD8 T cell pool inside the tumor, resulting in higher IFN-γ levels which trigger the increased expression of intratumoral antigen presenting molecules [20].

Currently, there are several published data in which RIPs are chemically linked to or genetically fused with antibodies, growth factors, hormones and lectins for the treatment of cancer. Based on the results of the present work, pulchellin may be a candidate for further studies as a drug that can produce immunotoxins with anti-tumoral purpose.

## Conclusions

Pulchellin exhibited several interesting activities, including macrophage activation, the production of cytokines known to increase antitumor immunity and the reduction of pro-tumoral cytokines and CD4^+^CD25^+^ Treg cells. These results provide a new perspective for cancer treatment and suggest that pulchellin may be a candidate for the production of immunotoxins against breast cancer.

## Abbreviations

FCS: Fetal calf serum; IC50: Inhibitory concentration 50%; IFN- γ: Interferon-gamma; IL-4: Interleukin-4; IL-6: Interleukin-6; IL-10: Interleukin-10; TGF-β: Transforming growth factor-beta; TNF-α: Tumor necrosis factor-alpha; Treg cells: Regulatory T cells.

## Competing interests

The authors declare that they have no competing interests.

## Authors’ contributions

IZC conceived the study, participated in its design and coordination. DCM carried out cell culture experiments, cytokine estimations and drafted the manuscript. LCAB participated of the immunoassays. MCPP and LFS realized the tumor induction. All authors read and approved the final manuscript.

## Pre-publication history

The pre-publication history for this paper can be accessed here:

http://www.biomedcentral.com/1472-6882/12/107/prepub
